# *Foxb1* Regulates Negatively the Proliferation of Oligodendrocyte Progenitors

**DOI:** 10.3389/fnana.2017.00053

**Published:** 2017-07-05

**Authors:** Yuanfeng Zhang, Elti Hoxha, Tianyu Zhao, Xunlei Zhou, Gonzalo Alvarez-Bolado

**Affiliations:** ^1^Department of Neuroanatomy, University of HeidelbergHeidelberg, Germany; ^2^Department of Urology, The 2nd Affiliated Hospital of Chongqing Medical UniversityChongqing, China; ^3^Key Laboratory of Oral Disease and Biomedical Sciences, Stomatological Hospital, Chongqing Medical UniversityChongqing, China

**Keywords:** Claudin11, GalC, lineage analysis, medulla oblongata, neuroepithelium, NG2, thalamus

## Abstract

Oligodendrocyte precursor cells (OPC), neurons and astrocytes share a neural progenitor cell (NPC) in the early ventricular zone (VZ) of the embryonic neuroepithelium. Both switch to produce either of the three cell types and the generation of the right number of them undergo complex genetic regulation. The components of these regulatory cascades vary between brain regions giving rise to the unique morphological and functional heterogeneity of this organ. *Forkhead b1 (Foxb1)* is a transcription factor gene expressed by NPCs in specific regions of the embryonic neuroepithelium. We used the mutant mouse line *Foxb1-Cre* to analyze the cell types derived from *Fobx1*-expressing NPCs (the *Foxb1* cell lineage) from two restricted regions, the medulla oblongata (MO; hindbrain) and the thalamus (forebrain), of normal and *Foxb1*-deficient mice. *Foxb1* cell lineage derivatives appear as clusters in restricted regions, including the MO (hindbrain) and the thalamus (forebrain). *Foxb1*-expressing NPCs produce mostly oligodendrocytes (OL), some neurons and few astrocytes. *Foxb1*-deficient NPCs generate mostly OPC and immature OL to the detriment of neurons, astrocytes and mature OL. The axonal G-ratio however is not changed. We reveal *Foxb1* as a novel modulator of neuronal and OL generation in certain restricted CNS regions. *Foxb1* biases NPCs towards neuronal generation and inhibits OPC proliferation while promoting their differentiation.

## Introduction

Oligodendrocytes (OL) produce and maintain the myelin sheaths around axons of the central nervous system (CNS). In addition, OL can also provide trophic support to axons and promote their viability (Emery, 2010; Mitew et al., 2014). Defects in myelination, the process by which OL wrap axons in layers of myelin, cause severe pathological conditions in humans. In addition, pathological conditions affecting the maturation of the brain white matter in premature infants are on the rise, and their most common result is mental retardation. Since myelination occurs mostly during CNS development, much research has focused on the development of OL. In particular, the mechanisms regulating the proliferation and differentiation of OL, the cells responsible for the formation of the white matter, are currently under intense scrutiny (Goldman and Kuypers, 2015; Marinelli et al., 2016).

OL generate from bipolar, migratory oligodendrocyte progenitor cells (OPC), probably heterogeneous in morphology and physiology, that arise from specific zones of the neuroepithelium ventricular zone (VZ). OPC are identified by the expression of a series of specific antigens, including NG2 chondroitin sulfate proteoglycan (NG2), platelet-derived growth factor-alpha receptor (PDGFRα) and oligodendrocyte transcription factor 2 (Olig2). The OPC specifically express a number of transcription factors as they migrate to colonize the CNS and produce OL. Each phase in the development of OL (OPC, premyelinating OL and myelinating OL) is associated with intense expression of specific sets of transcription factors; once at the final destination, OL mature and acquire the expression of specific markers: MBP, PLP, MAG and Claudin11 among others and acquire their typical morphology as well as the ability to myelinate (Noll and Miller, 1993; Rowitch and Kriegstein, 2010; de Castro et al., 2013; Goldman and Kuypers, 2015). A key point in this pathway, the decision by the OPC to stop proliferating and start producing OL, is under strong control (Dugas et al., 2010).

There are different dorsal and ventral VZ specific areas generating OPC in the hindbrain (Davies and Miller, 2001; Vallstedt et al., 2005). Here we report expression of transcription factor gene *Forkhead b1 (Foxb1)* in one of the ventral OPC-generating regions in the hindbrain. *Foxb1* belongs to a gene family encoding hundreds of transcription factors whose DNA binding domain has a winged helix configuration (Weigel and Jäckle, 1990; Carlsson and Mahlapuu, 2002). *Foxb1* is expressed widespread in the early developing VZ of the neural tube and is later restricted to areas of the spinal cord, hindbrain, thalamus and hypothalamus (Ang et al., 1993; Kaestner et al., 1993, 1996; Alvarez-Bolado et al., 1999; Zhao et al., 2007). Involvement of *Foxb1* in OL development has not been reported.

Here we use a *Foxb1-Cre* knockin-knockout mouse line together with reporter mouse lines Z/AP and ROSA26R in order to approach the following questions: (1) Which specific cell types of the CNS are generated by *Foxb1*-expressing VZ (i.e., what is the *Foxb1* cell lineage in the brain)? (2) What is the role of *Foxb1* in oligodendrocyte development? Our results show that *Foxb1* is a novel player in OL development, whose role involves inhibiting OPC proliferation and promoting oligodendrocyte maturation.

## Materials and Methods

### Mouse Handling

All mouse lines were housed and fed according to the German Animal Welfare Act (Tierschutzgesetz) and the European Communities Council Directive in the Interfacultary Biomedical Facility (IBF), University of Heidelberg. The authorization for collecting mice brain and handling animal samples was approved by the Regierungspraesidium Karlsruhe, Baden-Wuerttemberg. The experiments were carried out in the Neuroanatomy Department, University of Heidelberg and all the procedures were performed according to the Animal Welfare Act. Adult mice were killed by cervical dislocation. The staining protocol for some antibodies required mice to be anesthetized with isofluorane and then intracardially perfused with fixative. For embryos, pregnant mice were sacrificed by cervical dislocation and E12.5, E15.5 or E18.5 pups were taken out of the uterus and processed for histology.

### Mutant Mouse Lines

#### Foxb1-Cre Mouse Line

In this mouse line, transcriptional activation of the *Foxb1* locus initiates expression of *Cre* recombinase and enhanced green fluorescent protein (*EGFP*; as well as *Foxb1* itself in heterozygotes; Zhao et al., 2007, 2008).

#### Z/AP Reporter Mouse Line

In *Foxb1^Cre^ x Z/AP* mice, *Foxb1*-expressing cells and their progeny permanently express human placental alkaline phosphatase (hPLAP; Lobe et al., 1999). hPLAP is a GPI-linked cell surface marker. In this reporter line, cells not expressing Cre recombinase are labeled by expression of *β-galactosidase* (negative control).

#### ROSA26R Mouse Reporter Line

When dealing with antibodies detecting proteins enriched in the cell nucleus (for instance, transcription factors, like Olig2), the use of a lineage reporter also expressed in the cell nucleus makes it easier to ascertain cellular colocalization of both marker and reporter. ROSA26R (Soriano, 1999) is such a reporter mouse line, carrying β-galactosidase as reporter gene; upon Cre recombination, antibody detection of β-galactosidase produces a characteristic punctate pattern in the cell nucleus (Soriano, 1999). In *Foxb1^Cre^ x ROSA26R* mice the presence of β-galactosidase in the nucleus of Cre-expressing cells can be detected from E8.5 on (Zhao et al., 2007, 2008).

### Labeling Alkaline Phosphatase Activity

Phosphatase/NBT staining has been described (Lobe et al., 1999; Gierut et al., 2014). Briefly, brains or embryos were dissected in PBS buffer on ice; fixed in 4% paraformaldehyde solution with 0.02% NP-40 and 0.01% sodium deoxycholate at 4°C for 30 min; washed in PBS three times for 30 min at 4°C; incubated in PBS at 72°C for 30 min to inactivate endogenous alkaline phosphatase; rinsed three times in PBS for 10 min at room temperature; washed in alkaline phosphatase buffer two times 10 min; stained with 100 mg/ml NBT and 50 mg/ml BCIP in AP buffer at 4°C until optimal results appeared; washed in PBS extensively to reduce the background; mounted in Mowiol.

### Immunohistochemistry

To detect specific cell markers as well as reporter proteins hPLAP and β-galactosidase, the following antibodies and conditions were used: anti-hPLAP (Sigma A2951) 1:2000 on paraffin sections (PS) or fixed frozen tissue (FFT); anti-β-galactosidase (Abcam ab9361) 1:200 (FFT); anti-Claudin11 (Abcam ab53041) 1:500 (FFT); anti-GalC (Chemicon AB142) 1:100 (PS); anti-myelin basic protein MBP (Sigma M3821) 1:100 (FFT); anti-platelet derived growth factor receptor alpha (PDGFRα; BD Pharmingen 558774) 1:300 on fresh frozen tissue; anti-NG2 (Chemicon AB5320) 1:100 (PS); anti-Olig2 (Chemicon AB9610) 1:200 (FFT); anti-GFAP (Chemicon AB5804) 1:300 (FFT); anti-β-tubulin III (Abcam ab18207) 1:600 (FFT); anti-NeuN (Abcam ab177487) 1:500 (FFT).

### Labeling of Thalamic Neurons through Transfection with Two-Reporter Construct

We cloned a DNA construct carrying a constitutive GFP reporter and a Cre-recombination-dependent tdTomato reporter. Every cell transfected will express the green GFP reporter, but transfected cells expressing Cre will express the red tdTomato reporter instead. Our construct was based on MSCV FLIPi P2G_Thy1.1 Dbl (p53, PTEN), a gift from Richard Hynes (Addgene plasmid #19746; Stern et al., 2008). We introduced the following modifications for our purposes: (1) removal of the cassette Puromycin 2a-GFP-Thy1.1-miR-WRPE; (2) insertion of the reporter tdTomato in reverse orientation in the place of Thy1.1-miR; (3) addition of the CAG promoter. The resulting DNA construct was transfected into the thalamus of E12.5 wild type mice through *in utero* electroporation. The electroporation technique has been described before (Tabata and Nakajima, 2001; Haddad-Tóvolli et al., 2013).

### Proliferation Assay

For proliferation studies, 50 mg/kg of body weight of Bromo-deoxy-uridine (BrdU) was intraperitoneally injected as aqueous solution into P10 mice three times, at 4 h intervals (Bu et al., 2004). Mice were sacrificed 2 h after the last injection. The brains were dissected and fixed in 4% PFA overnight at 4°C, washed in PBS for 4–6 h, cryoprotected in 0.1 M PBS containing 30% sucrose, embedded in OCT compound on Dry Ice and stored at −80°C. Previous to immunodetection of BrdU and Olig2, cryosections (20 μm) were incubated in 1 M HCl for 10 min on ice and then in 2 M HCl for 30 min at 37°C and neutralized with 0.1 M sodium borate buffer at room temperature.

### Confocal Microscope

The results were observed and analyzed under a confocal laser-scanning microscope (Zeiss LSM 510) with ZEN 2010 software.

### Electron Microscopy and G-Ratio

The G-ratio was calculated on measurements performed on electron microscopy photographs of transverse sections of medulla oblongata (MO). The tissue samples were obtained from *Foxb1^Cre/+^* and *Foxb1^Cre/Cre^* mouse brains (age P56, three mice per genotype), then processed according to current electron microscopy protocols. The diameter of 80 randomly chosen axons and the corresponding fibers (axon + myelin) were measured for each individual mouse. The data were analyzed with the Mann-Whitney test.

### Statistical Analysis

Cell countings were performed on 20 μm sections from the brains of three animals per genotype. Sagittal sections were cut in four series (A, B, C and D) from an area spanning the middle (rostro-caudally) of the hindbrain. Eight sections were counted per mouse. The counting bin for marker-labeled cells was 600 μm × 600 μm in MO. The counting bin for Olig2 plus BrdU was 0.1 mm^2^ in MO. The localization of the bins was chosen randomly. Statistical assessment was performed with Prism 5 software (GraphPad Software, San Diego, CA, USA). The Mann-Whitney test was used and the results are represented in bar graphs with mean ± SD; *p < 0.05, **p < 0.01, ***p < 0.005.

## Results

### *Foxb1* Is Expressed in Oligodendrocyte- Generating Regions of the Embryonic Hindbrain

**Figure 1:**
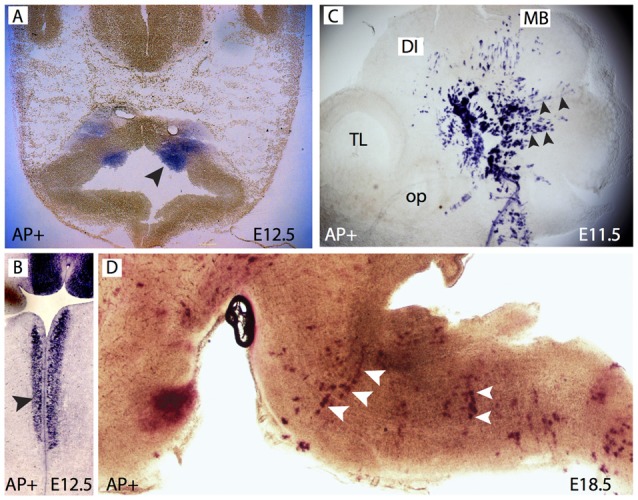
*Forkhead b1 (Foxb1)* lineage cells in the brainstem neuroepithelium and mantle layer.** (A)** Histochemical demonstration of restricted AP activity detection in the ventral medullary neuroepithelium (arrowhead) of the E12.5 *Foxb1^Cre/+^ x Z/AP* mouse. **(B)** Similar to **(A)** on a longitudinal section. **(C)** AP activity on a whole mount E11.5 *Foxb1^Cre/+^ x Z/AP* mouse showing radially migrating cells (arrowheads). **(D)** AP activity labels rows of radially oriented glial-like cells in the pons and medulla of an E18.5 *Foxb1^Cre/+^ x Z/AP* mouse (sagittal section).

*Foxb1* is expressed in two narrow longitudinal bands in the VZ of the brainstem (Alvarez-Bolado et al., 1999) reminiscent of the domains of expression of OPC marker genes *Olig1* and *Olig2* (Zhou et al., 2000). We asked if the lineage of the *Foxb1*-expressing neuroepithelium included OL and if *Foxb1* was involved in oligodendrocyte development. We approached this question by using a lineage-labeling mouse line, the *Foxb1-Cre-EGFP x Z/AP*. *Foxb1-Cre-EGFP* is a knockin-knockout of the Cre recombinase and the expression reporter EGFP into the *Foxb1* locus (Zhao et al., 2007). By crossing these lines with the Z/AP reporter line (Lobe et al., 1999), *Foxb1*-expressing neuroepithelial cells as well as their entire progeny (even if they do not express *Foxb1*) will permanently express hPLAP in this way becoming identifiable. First, we examined the brains of mouse embryos heterozygous for this insertion. *Foxb1*-heterozygotes have normal phenotype (Dou et al., 1997; Alvarez-Bolado et al., 2000; Kloetzli et al., 2001) and were used here as proxies for the wild type. Treating early embryonic brains with a procedure to detect alkaline phosphatase activity, we detected as expected the bands in the hindbrain (Figures 1A–C). Later in development, we detected AP+ cells in the MO (Figure 1D).

**Figure 2:**
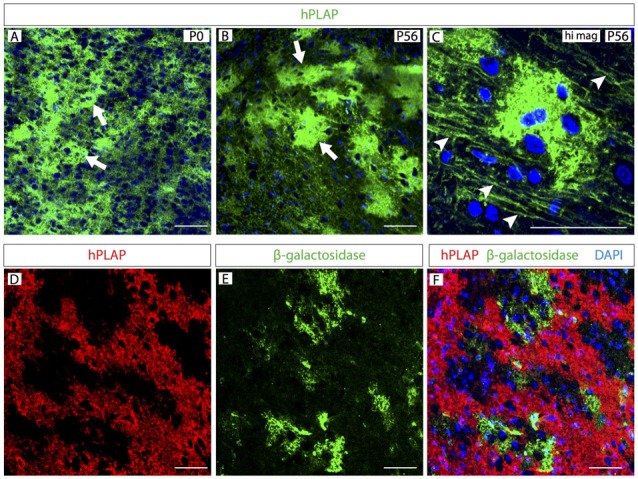
Clusters of *Foxb1*-lineage cells in the postnatal brainstem. **(A–C)** Human placental alkaline phosphatase (hPLAP) detection by antibodies on sections of *Foxb1^Cre/+^ x Z/AP* mouse brainstem. Cell clusters (arrows in **A,B**) expressing hPLAP become increasingly more distinct during the first 2 months of age. Additionally, arrowheads in C show labeled axon-like structures. **(D–F)** hPLAP (red in **D,F**) and β-galactosidase (green in **E,F**) detection by antibodies on sections of *Foxb1^Cre/+^ x Z/AP* mouse brainstem. The hPLAP-labeled tissue does not overlap with β-galactosidase-expressing regions. Scale bars 50 μm.

By staining MO sections of these mice for hPLAP, we labeled large groups of cells with multiple processes in brains of every developmental age (Figure 2). As development proceeds, *Foxb1*-lineage cells form clusters in the brainstem as well as axon-like structures (Figures 2A–C). An internal control is built in the reporter mouse *Z/AP* (Lobe et al., 1999), so that cells not undergoing a Cre recombination (i.e., not expressing the *Cre* driver, in this case *Foxb1*) express β-galactosidase as a reporter instead. We labeled expression of both markers on brainstem sections to confirm that the hPLAP-expressing clusters did not overlap with β-galactosidase-expressing regions (Figures 2D–F).

### The *Foxb1* Lineage Includes Neurons as well as Large Numbers of Oligodendrocytes

**Figure 3:**
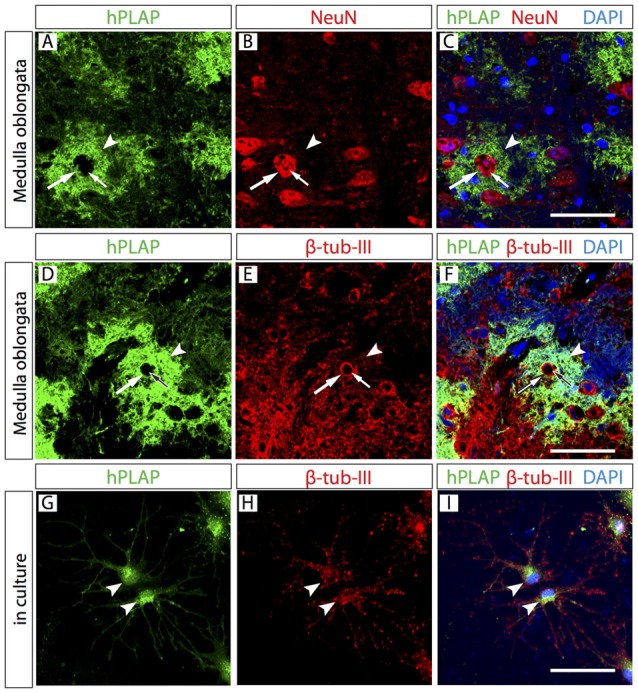
Neurons are present in the *Foxb1*-lineage cell clusters. Antibody detection of hPLAP, NeuN and β-tubulin-III (as indicated) on medulla oblongata (MO) sections of P56 *Foxb1^Cre/+^ x Z/AP* mouse **(A–F)** and on primary culture of P56 hindbrain **(G–I)**. **(A–C)** NeuN-labeled cell body (large arrow) and cell nucleus (small arrow) inside a hPLAP-expressing cluster (arrowhead). **(D–F)** β-tubulin-III-expressing cell body (large arrow) and its cell nucleus (small arrow) inside a hPLAP-expressing cluster (arrowhead). **(G–I)** Antibody detection of hPLAP and β-tubulin-III (as indicated) on neurons in culture. Scale bars 50 μm.

In order to identify the cellular components of these clusters, we colocalized hPLAP with neuron-specific markers (either β-tubulin-III or NeuN) on *Foxb1^Cre/+^ x Z/AP* brains and found a number of double-labeled cells (Figures 3A–F). We confirmed this on primary culture of *Foxb1^Cre/+^ x Z/AP* brain cells (Figures 3G–I). Colocalizing astrocyte-specific protein glial fibrillary acidic protein (GFAP) with hPLAP also showed a small number of *Foxb1*-lineage astrocytes (not shown). As expected, no hPLAP-labeled cells expressed microglial marker Iba-1, since microglia originates outside of the neural tube and therefore far from the *Foxb1*-expressing neuroepithelium (not shown).

**Figure 4:**
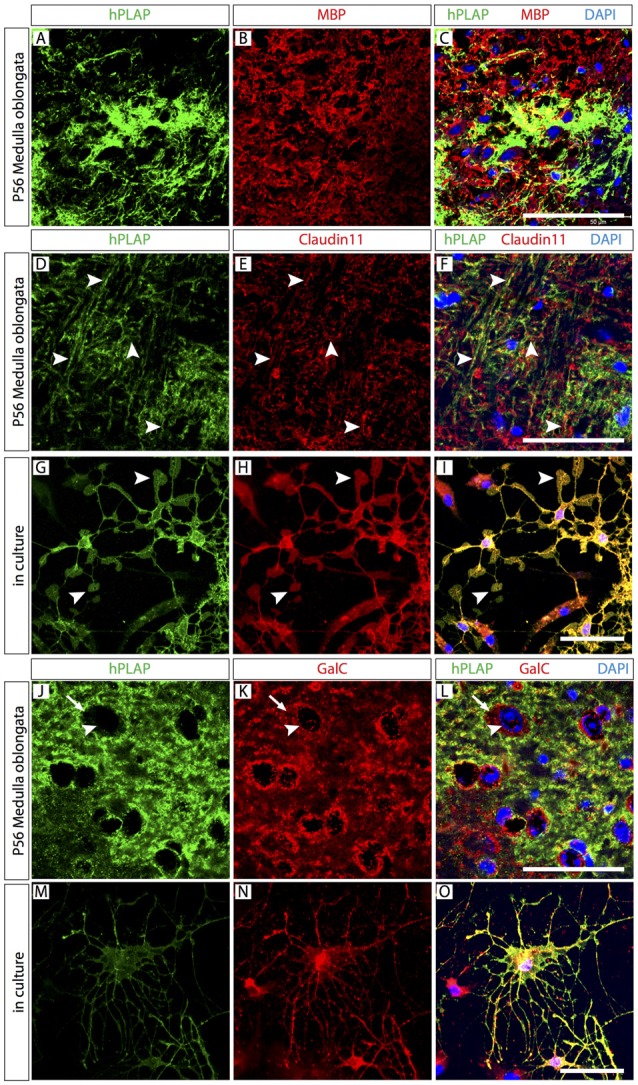
Abundant mature and immature oligodendrocytes (OL) in the *Foxb1* lineage. Antibody detection of hPLAP, myelin basic protein (MBP), Claudin11 and Galactocerebroside C (GalC) (as indicated) on MO sections or primary cell culture (as indicated) of P56 *Foxb1^Cre/+^ x Z/AP* mouse. **(A–C)** Colocalization of MBP and hPLAP. **(D–F)** Claudin 11 colocalizes with hPLAP-expressing axon bundles (arrowheads). **(G–I)** Claudin 11 colocalizes with hPLAP-expressing cultured brain cells with the morphology of mature OL (arrowheads indicate their typical lamella extensions). **(J–L)** Colocalization of GalC and hPLAP. Arrows indicate the boundary of the GalC-labeled cytoplasm; arrowheads indicate the corresponding cell nucleus. **(M–O)** GalC colocalizes with hPLAP-expressing cultured brain cells with the morphology of immature OL. Scale bars 50 μm.

The finding that only small numbers of hPLAP-labeled brain cells were neurons or astrocytes suggested that the most abundant cellular component of the *Foxb1* lineage could be OL. Expression of myelin basic protein (MBP) colocalized partially with hPLAP (Figures 4A–C), in principle confirming this impression. The appearance of the tissue was however complex and did not provide appropriate cellular resolution (Figures 4A–C). Expression of Claudin11, a marker of mature OL (Bronstein et al., 2000), colocalized with profiles morphologically similar to myelinated axons (Figures 4D–F). On primary cultures of cells from the MO of *Foxb1^Cre/+^ x Z/AP* mice, Claudin11 and hPLAP colocalized on cells with unequivocal mature oligodendrocyte morphology, including the characteristic lamellae (Figures 4G–I). Expression of Galactocerebroside C (GalC), a marker of mature as well as immature OL, also colocalized with hPLAP on sections (Figures 4J–L) and on primary cultures (Figures 4M–O).

### Abundant OPC Belong to the *Foxb1*-Lineage

**Figure 5:**
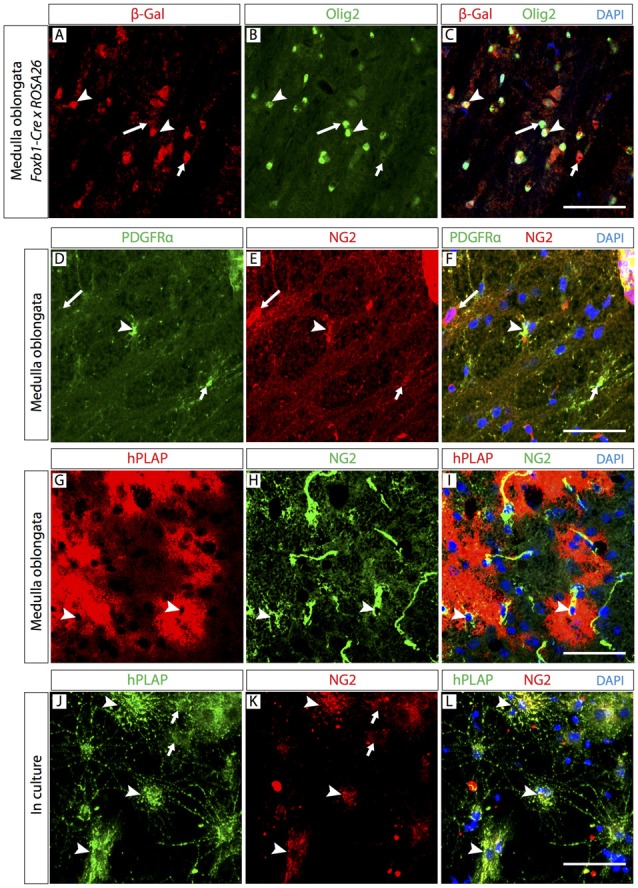
The *Foxb1* lineage includes oligodendrocyte precursor cells (OPC). Antibody detection of hPLAP, Olig2, NG2 and platelet-derived growth factor-alpha receptor (PDGFRβ; as indicated) on MO sections of P56 *Foxb1^Cre/+^ x ROSA26R* mouse **(A–C)** or P56 *Foxb1^Cre/+^ x Z/AP* mouse **(A–I)** or on primary culture of P56 MO **(J–L)**. **(A–C)** The *Foxb1*-lineage marker β-galactosidase colocalizes with a subpopulation of Olig2-expressing cells (arrowheads). Large and small arrows indicate cells expressing only Olig2 or only β-galactosidase, respectively. **(D–F)** Colocalization of OPC markers PDGFRβ and NG2 (arrowheads). Large and small arrows indicate cells expressing only NG2 or only PDGFRβ, respectively.** (G–I)** In hPLAP-labeled cell clusters (red), some cells express NG2 (arrowheads). **(J–L)** hPLAP colocalizes with NG2 on some brain cells (arrowheads) in primary culture. Arrows indicate non-colocalizing cells. Scale bars 50 μm.

If most of the observed *Foxb1*-lineage brain cells are OL, it follows that some OPC must also belong to this lineage. To confirm this, we colocalized *Foxb1*-lineage reporters with OPC markers Olig2 and NG2 (Figure 5). Since Olig2 protein localizes to the cell nucleus, in order to colocalize it with a reporter we crossed our *Foxb1^Cre/+^ x Z/AP* mice with the reporter mouse line ROSA26R (Soriano, 1999), whose cells express β-galactosidase as reporter upon Cre recombination. We found numerous cells expressing both this reporter and Olig2 (Figures 5A–C) in the MO of *Foxb1^Cre/+^ x ROSA26R* mice. The β subunit of the receptor for PDGFRβ as well as NG2 specifically colocalize in OPC (Richardson et al., 1988; Hart et al., 1989). We found also many cells where NG2 colocalized with hPLAP in histological sections of the MO (Figures 5G–I) as well as in culture (Figures 5J–L; see cell abundance quantitation below, Figure 8).

**Figure 6:**
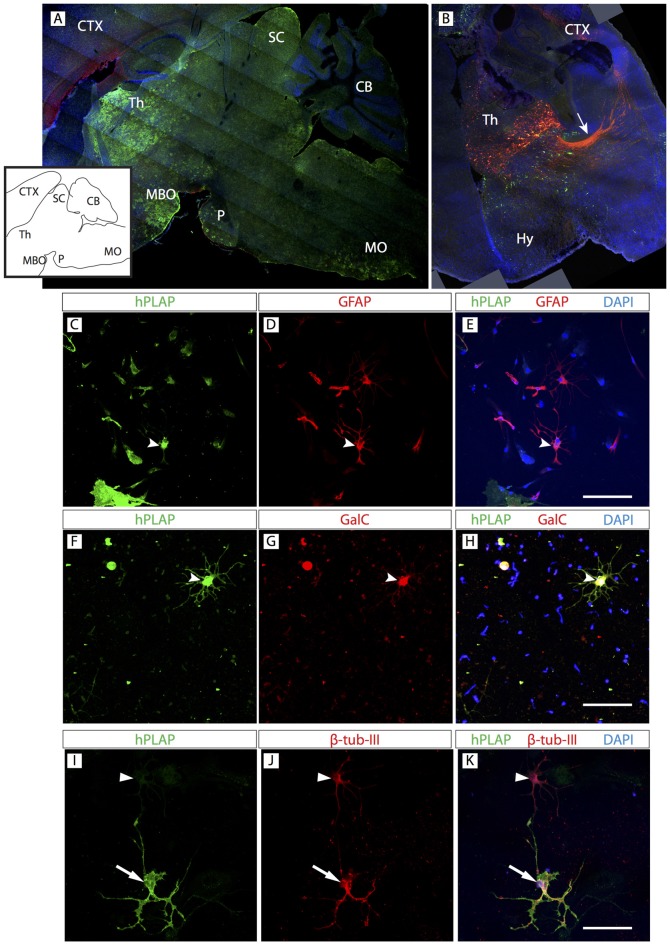
*Foxb1*-lineage clusters are also abundant in the thalamus.** (A)** Sagittal view of a P56 *Foxb1^Cre/+^ x Z/AP* mouse brain (rostral to the left) showing hPLAP-expressing clusters in thalamus (Th), mammillary body (MBO), superior colliculus (SC), pons (P) and MO. Inset: line profile of the section in **(A)**. **(B)** Transverse section of P56 *Foxb1^Cre/+^* after electroporation (at E12.5) and culture (see “Materials and Methods” Section for details). *Foxb1*-lineage thalamic neurons and cortico-thalamic axons (arrow) are labeled red. Transfected cells not expressing *Foxb1* are green. **(C–K)** Primary culture of *Foxb1^Cre/+^ x Z/AP* thalamus showing colocalization of hPLAP with GFAP **(C–E)**, GalC **(F–H)** and β-tubulin-III **(I–K)**. Scale bars 50 μm.

**Figure 7:**
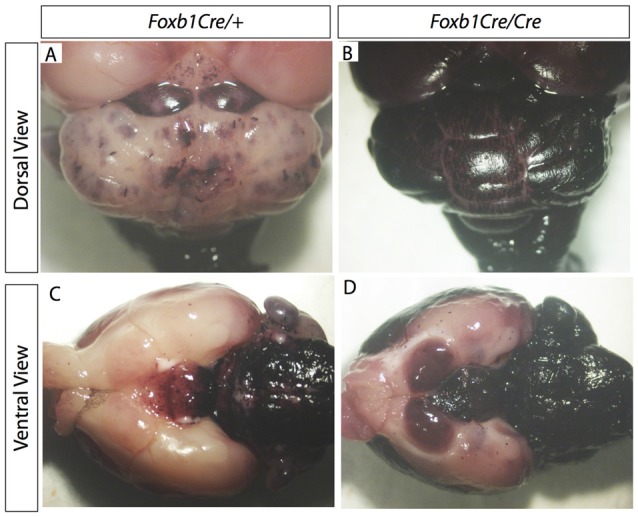
Increase in *Foxb1* lineage in the homozygous *Foxb1* mutant. Whole mount AP activity detection on P56 *Foxb1^Cre/+^ x Z/AP* **(A,C)** and *Foxb1^Cre/Cre^ x Z/AP* **(B,D)** brains.

**Figure 8:**
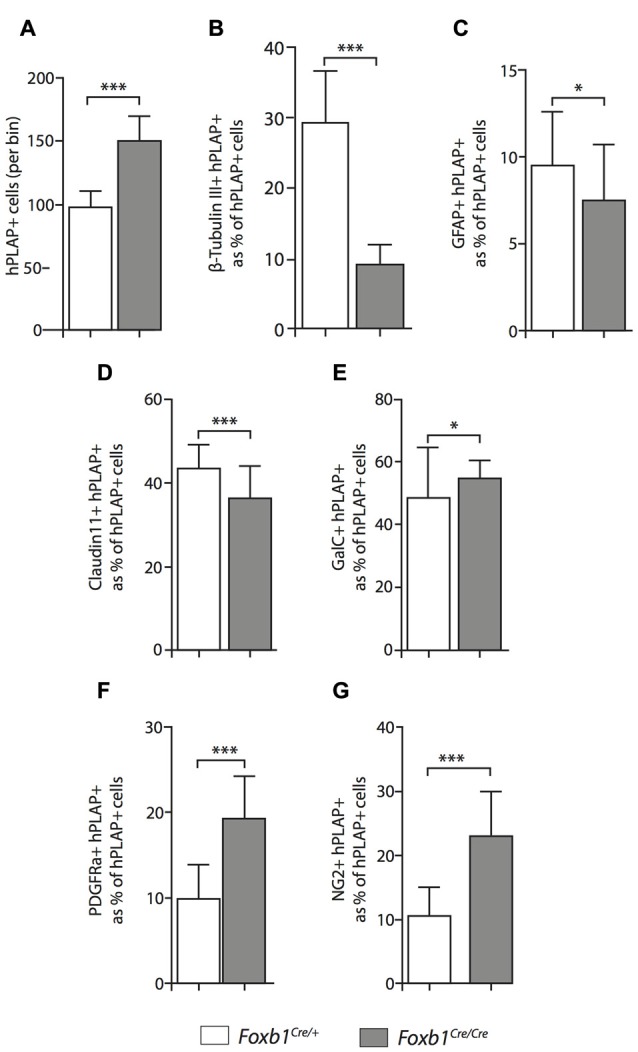
Differential changes in cell number between cell subpopulations in the *Foxb1*-deficient MO.** (A)** The absolute number of hPLAP-expressing cells increases in the *Foxb1^Cre/Cre^ x Z/AP* brain. **(B–G)** Number of cells coexpressing hPLAP and cell-type specific markers (as indicated) in the P56 *Foxb1^Cre/+^ x Z/AP* and *Foxb1^Cre/Cre^ x Z/AP* **(B,D)** MO. The results are represented as percent of all hPLAP-expressing cells. Neurons and astrocytes **(B,C)** decrease in the mutant, as do mature OL **(D)**. Cells expressing OPC markers PDGFRβ and NG2 **(F,G)** increase in number, as does the total number of mature plus immature OL **(E)**. Beware the different scales of the bar graphs. Mean ± SD; *p < 0.05, ***p < 0.005; Nonparametric Mann-Whitney test.

### The Thalamus Shows Similar *Foxb1*-Lineage Cell Clusters

OPC originate from certain spatially restricted regions of the neural tube. They then migrate and proliferate to supply the entire CNS with OL. A current question concerns the possibility that OL from different origins have differential properties and if OL from any given region can be used to substitute for OL in any other region of the brain (replacement therapy). In this context, we asked if the basic composition of the *Foxb1* lineage in the hindbrain was similar to that of another regionally restricted subpopulation of *Foxb1*-lineage brain cells situated not in the hindbrain but in the forebrain. The thalamus is a forebrain region with strong *Foxb1* developmental expression and a large part of the thalamus is of *Foxb1* lineage (Zhao et al., 2007, 2008).

**Figure 9:**
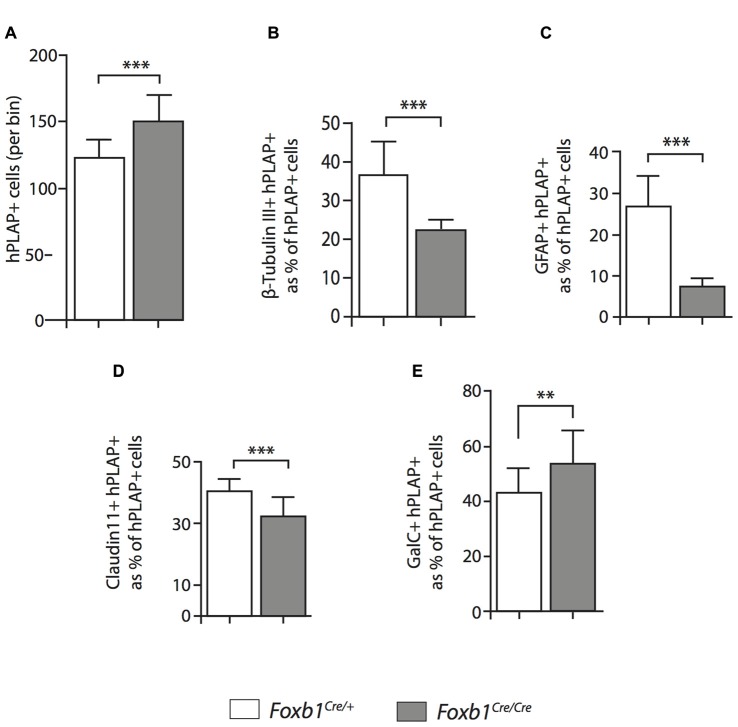
Differential changes in cell number between cell subpopulations in the *Foxb*-deficient thalamus. Number of cells coexpressing hPLAP and cell-type specific markers (as indicated) in the P56 *Foxb1^Cre/+^ x Z/AP* and *Foxb1^Cre/Cre^ x Z/AP* thalamus. The results are represented as percent of all hPLAP-expressing cells. The number of *Foxb1*-lineage cells increases in the mutant **(A)**. Neurons and astrocytes **(B,C)** decrease in the mutant, as do mature OL **(D)**. The total number of mature plus immature OL increases **(E)**. Mean ± SD; **p < 0.01, ***p < 0.005; Nonparametric Mann-Whitney test.

Clusters expressing hPLAP were obvious in the thalamus (Figure 6A). A characteristic property of the thalamus with important functional consequences is the presence of many neurons sending axonal projections to the cortex. We used *in utero* electroporation with a plasmid expressing upon Cre recombination the red reporter tdTomato (see “Materials and Methods” Section for details), which fills the cell body and axons. The results showed abundant *Foxb1-*lineage thalamo-cortical neurons (Figure 6B). We confirmed the presence of OL, neurons and astrocytes colocalizing hPLAP on primary thalamic culture (Figures 6C–K; see cell abundance quantitation below, Figure 9).

### The *Foxb1*-Lineage in *Foxb1* Mutant Mice Produces Abnormally Large Numbers of OPC

Comparison of *Foxb1^Cre/+^ x Z/AP* and *Foxb1^Cre/Cre^ x Z/AP* mouse brains after whole mount histochemical demonstration of AP activity showed very increased staining in the homozygotes (Figures 7A–D). This suggested that *Foxb1* had an influence on the cell abundance and maybe also identity. To investigate this, we performed cell countings on tissue of heterozygotes and homozygotes labeled with specific cell markers as well as hPLAP. Our results show, first, that in homozygotes there are more *Foxb1*-lineage cells (Figure 8A). We also found that, in homozygotes, *Foxb1*-lineage neurons, astrocytes and mature OL were reduced (Figures 8B–E). However, OPC were increased (Figures 8F,G). Interestingly, a first approach to the abundance of different *Foxb1*-lineage cell types in the thalamus yielded similar results (Figures 9A–D). This suggests that all *Foxb1*-lineage OL are functionally homogeneous.

**Figure 10:**
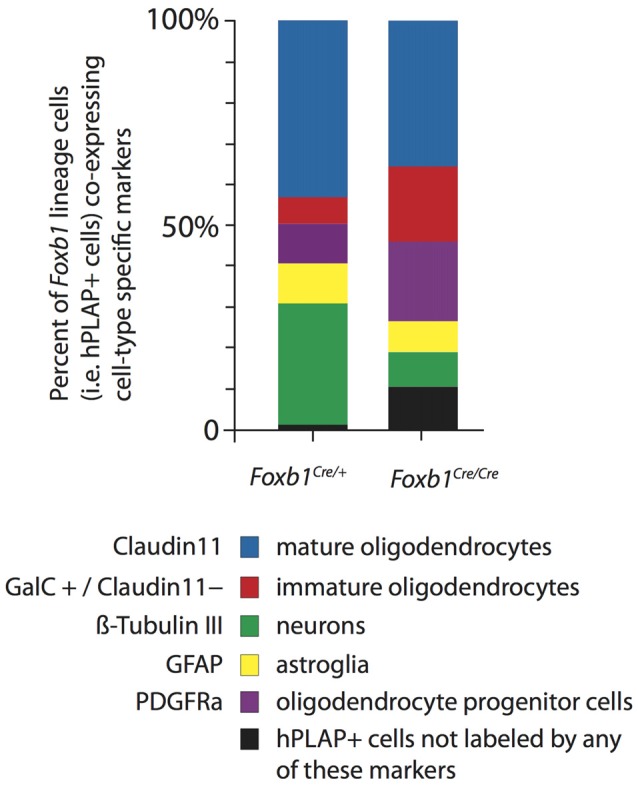
Increase in OPC and immature OL to the expense of other cell types in the *Foxb1*-deficient MO. Abundance of different types of *Foxb1* lineage cells in P56 *Foxb1^Cre/+^ x Z/AP and Foxb1^Cre/Cre^ x Z/AP* as percent of all Foxb1 lineage cells (i.e., all hPLAP cells). In the homozygous mutant there is an increase in OPC and immature OL and an important decrease in the number of neurons and mature OL.

Although the exact proportion of *Foxb1*-lineage cells to other brain cells is not central to our study, we thought it was of some interest to obtain at least a rough approximation to this value for neurons, OL and OPC. Therefore we counted non-hPLAP (i.e., non-*Foxb1*-lineage) cell in the bins used to quantitate the hPLAP-expressing (*Foxb1*-lineage) cells. The results are as follows: (1) mature plus immature OL (i.e., GalC-expressing cells) belonging to the *Foxb1*-lineage are 73.17% of the total GalC-positive cells per bin in heterozygotes, and 90.00% in homozygotes; (2) OPC (i.e., NG2-expressing cells) belonging to the *Foxb1*-lineage are 45.40% of all NG2-positive cells per bin in heterozygotes and 75.89% in homozygotes; and (3) neurons (i.e., cells expressing beta-tubulin III) belonging to the *Foxb1*-lineage are 30% of all beta-tubulin III-expressing cells per bin in heterozygotes and 24.78% in homozygotes. As could be expected, OL and OPC of the *Foxb1* lineage “crowd out” the non-*Foxb1* lineage ones in homozygotes, while the proportion of *Foxb1*-lineage neurons becomes slightly smaller. These data however cannot be directly compared to our cell countings summarized in Figure 10, since those are represented in percent of hPLAP-expressing cells (as opposed to percent of all cells in the bin).

We concluded that, in the *Foxb1*-deficient MO, the OPC increase to the expense of neurons and mature OL (Figure 10).

### OPC Proliferation Increased in *Foxb1* Homozygotes

**Figure 11:**
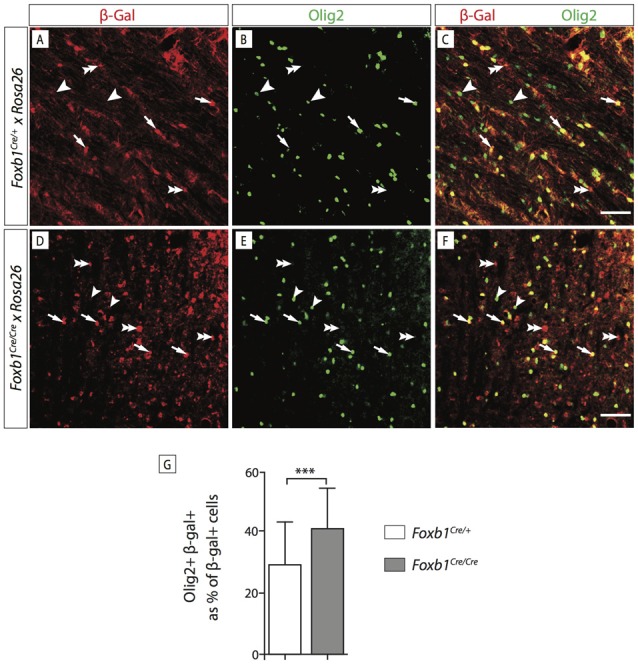
*Foxb1* inactivation increases the number of *Olig2*-expressing cells.** (A–F)** Antibody detection of β-galactosidase and Olig2 on the MO of P56 *Foxb1^Cre/+^ x ROSA26R* and **(A–C)** *Foxb1^Cre/Cre^ x ROSA26R* **(D–F)** mice as indicated. Cells expressing Olig2 and β-galactosidase can be seen (arrows) as well as cells expressing only Olig2 (arrowheads) or only β-galactosidase (double arrowheads). Scale bars 50 μm. **(G)** In homozygotes, the *Foxb1* lineage shows many more Olig2-expressing cells than in heterozygotes. Mean ± SD; ***p < 0.005; Nonparametric Mann-Whitney test.

**Figure 12:**
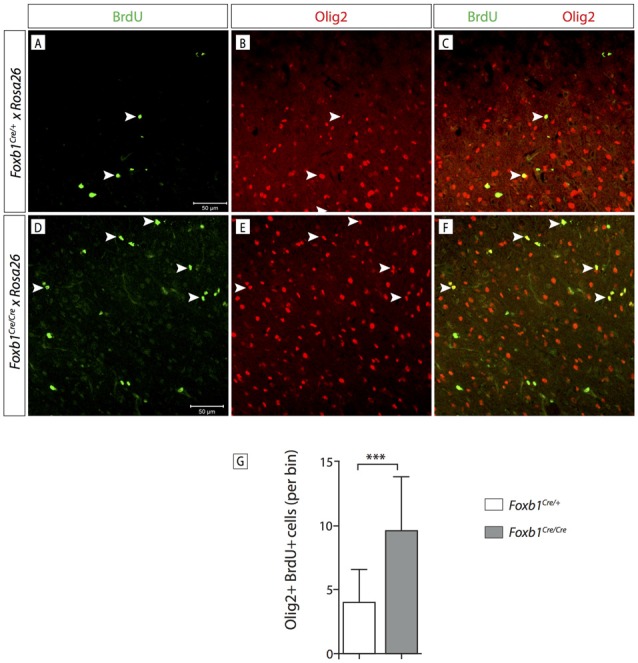
*Foxb1* inactivation increases the proliferation of *Olig2*-expressing cells.** (A–F)** Antibody detection of BrdU and Olig2 on the MO of P56 *Foxb1^Cre/+^* and **(A–C)** *Foxb1^Cre/Cre^* **(D–F)** mice as indicated. Arrowheads show some examples of double-labeled cells. **(G)** Significantly more Olig2 cells incorporated BrdU in Foxb1 homozygotes than in heterozygotes. Mean ± SD; ***p < 0.005; Nonparametric Mann-Whitney test.

In order to confirm this increase in OPC, we wanted to use another OPC marker, Olig2. Since Olig2 is localized in the nucleus, we wanted a reporter of the *Foxb1* lineage also localized in the nucleus. For this reason we used a different reporter, the ROSA26R mouse line (Soriano, 1999). In this line, Cre recombination causes expression of β*-galactosidase* which can be seen in the nucleus. We colocalized Olig2 and β-galactosidase on MO sections of heterozygotes and homozygotes (Figures 11A–F). Cell counting confirmed a significant increase of Olig2+ cells in the homozygotes (Figure 11G). Additionally, we labeled dividing cells with BrdU (see “Materials and Methods” Section for details) and then labeled the tissues with BrdU and Olig2 (Figure 12). We found that, in homozygous mutants, more Olig2-expressing cells were dividing than in heterozygotes.

### The G-Ratio Is Not Altered in the *Foxb1*- Deficient Hindbrain

**Figure 13:**
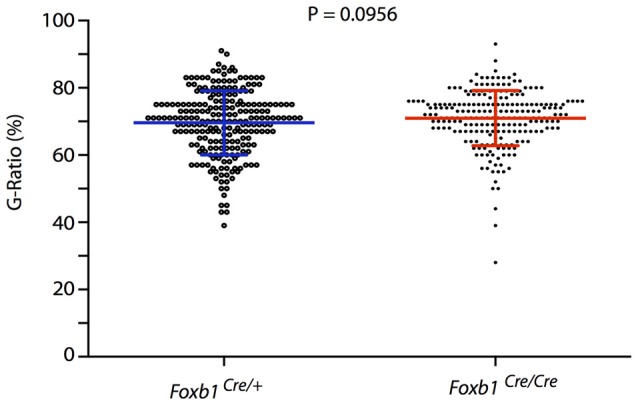
No change in G-ratio in the *Foxb1*-deficient MO. Mean ± SD for the *Foxb1^Cre/+^* 69.1 ± 9.544; for the *Foxb1^Cre/Cre^* 70.98 ± 8.202. Mann-Whitney test, *P* = 0.0956.

Finally, we asked if there would be any obvious defect in myelination in the hindbrain of our mutants. The phenotype of *Foxb1*-deficient mice includes several behavioral and CNS developmental defects (Dou et al., 1997; Labosky et al., 1997; Alvarez-Bolado et al., 2000; Radyushkin et al., 2005; see “Discussion” Section). However, alterations suggesting myelination defects have not been described in these mice. This suggests that the OL in this mutant are able to myelinate correctly. To test this hypothesis, we measured axon and fiber diameter on electron microscopy images from the hindbrain of homo- and heterozygotes and found that the G-ratio (Friede and Bischhausen, 1982) does not change in the mutants (Figure 13).

## Discussion

Lineage analysis of a mouse mutant line reveals transcription factor *Foxb1* as a novel player in oligodendrocyte development, promoting neuron generation and OPC differentiation over OPC generation and proliferation.

This novel function of *Foxb1* is in line with the reported functions of the Fox superfamily of transcription factors, which regulate genetic cascades controlling proliferation, differentiation, metabolism, aging, survival and apoptosis (Carlsson and Mahlapuu, 2002). A case in point is the Foxo subfamily, consisting of negative regulators of genes crucial for cell cycle progression (Lam et al., 2013). *Foxb1* is reportedly essential for the survival of a specific subpopulation of spinal motoneurons (Dou et al., 1997) and of a subset of hypothalamic cells (Alvarez-Bolado et al., 2000). However, a relation of *Foxb1* with OL or the control of cell proliferation has not been described before.

In the *Foxb1* null mutant mouse, the OL lineage increases to the detriment of the neuronal lineage. All neurons and macroglia share an early progenitor in the VZ of the embryonic neuroepithelium (Battiste et al., 2007; Pinto and Götz, 2007; Kriegstein and Alvarez-Buylla, 2009). Since part of the neuroepithelium belongs to the *Foxb1* lineage (i.e., expresses *Foxb1* or derives from *Foxb1*-expressing neural progenitor cells, NPCs), we have to assume that *Foxb1*-deficient NPCs are biased towards OPC generation. Therefore, a general function (direct or indirect) of *Foxb1* would be to modulate a complex genetic cascade which has been given the name of neurogenesis/gliogenesis switch (Martynoga et al., 2012). In the cortex, the most extensively researched CNS region, neurogenesis requires Fox transcription factor genes like *Foxc1* and *Foxg1*; reviewed in Martynoga et al. (2012). *Foxb1*, not expressed in the cortical VZ, could fulfill a similar role in other neuroepithelial regions.

Another consequence of *Foxb1* deficiency is the predominance of OPC and immature OL over mature OL, indicating an additional function of *Foxb1* inhibiting OPC proliferation and promoting their differentiation. Other members of the Forkhead gene family have roles in OL development. *Foxj3* seems to have the opposite function to Foxb1, by stimulating OPC proliferation (Dugas et al., 2010). *Foxa2* is essential for the induction and/or differentiation of OL in zebrafish (Norton et al., 2005) and *Foxg1* inhibits the glial fate choice (including oligodendroglia; Brancaccio et al., 2010).

Our results suggest that *Foxb1* would be one of many components in a cascade of genetic activation regulating a series of choices made by NPCs and the OPCs that derive from them. This function of *Foxb1* would be exerted in the spatially restricted groups of NPCs expressing this gene, the ones giving rise to cell groups in the hindbrain and thalamus (and possibly also in the mammillary body (MBO) of the hypothalamus). Because other Fox genes are involved in similar functions in the cortex, we can assume that, to a degree, different restricted regions of neuroepithelium regulate the generation of the corresponding brain cells by using different members of the same gene families. Each of these members might perform a similar job but with some differences upon which the morphological and functional heterogeneity of CNS regions in the adult would ultimately be based.

*Foxb1* is widely expressed in the neuroepithelium of the thalamus, hypothalamus, hindbrain and spinal cord (Alvarez-Bolado et al., 1999; Zhao et al., 2007, 2008). Accordingly, we have observed clusters of *Foxb1* lineage in thalamus, MBO (hypothalamus), pons and MO (we did not investigate the spinal cord) but not, for instance, in the cortex (Figure 6A). Therefore, here we uncover specific, spatially restricted subpopulations of OPC and OL characterized by belonging to the *Foxb1* lineage and whose proliferation is modulated by *Foxb1*. In the rodent CNS, OPC originate in discrete loci of the dorsal and ventral neuroepithelium (Woodruff et al., 2001; Rowitch, 2004; Kessaris et al., 2006; Rowitch and Kriegstein, 2010; Huang et al., 2013). Origin heterogeneity has some correlate in cellular properties like proliferation (Young et al., 2013) and marker expression (Butt et al., 1998b; Kleopa et al., 2004). Besides, different morphological classes of OL (Bjartmar et al., 1994; Butt et al., 1998a; Anderson et al., 1999) have been described, perhaps related to place of origin.

To address the question of the functional equivalence of OL of heterogeneous origins, we compared the main characteristics of lineage and phenotype between MO and thalamus. In both regions the *Foxb1* lineage consists mostly of neurons and OL, together with some astrocytes. Interestingly, a robust portion of the thalamo-cortical bundle tract was labeled by our reporter (Figure 6A), indicating that a large number of thalamo-cortical projection neurons belong to the *Foxb1* lineage. The *Foxb1* null phenotype is similar in both regions: reduction of neurons, astrocytes and mature OL in favor of immature OL. In principle, this agrees with previous work indicating that OL from OPC originated in different CNS regions are not very different functionally (Kessaris et al., 2006; Psachoulia et al., 2009; Tripathi et al., 2011; Clarke et al., 2012; although white matter and gray matter OL are functionally different from each other; Viganò et al., 2013). But the two subpopulations that we have compared share *Foxb1* lineage. Obviously OL from non-*Foxb1*-expressing regions will express different proliferation modulators. This implies that there must be a series of restricted neuroepithelial “patches” differentially expressing transcription factors which specifically modulate aspects of OL production.

Finally, a myelination phenotype could in principle be expected in the Foxb1 mouse mutants. However, neurological signs demyelination (like ataxia, unsteady gait, ocular paralysis, weakness and loss of sensation) have not been observed by us or other authors in these mutants. In addition, the G-ratio remained unchanged in the homozygous brainstem. We conclude that *Foxb1* is not required for normal myelination.

## Conclusion

Forkhead transcription factor *Foxb1* is a novel modulator of neuronal and OL generation acting in certain restricted CNS regions. *Foxb1* biases NPCs towards the neuronal and to a lesser degree the astrocytic lineages and inhibits the proliferation of OPC, favoring their differentiation. *Foxb1*-lineage OL are a novel subpopulation whose specific properties as well as their ability to replace other subpopulations (for instance, telencephalic OL) as part of a therapeutic approach are open questions.

## Author Contributions

YZ and EH performed experiments and analyzed results; TZ made the original observations and performed key preliminary experiments; XZ generated the *Foxb1-Cre* mutant line, assisted with the experiments and analyzed results; GA-B and XZ directed the project; YZ, EH and GA-B wrote the article; all authors revised the manuscript and approved the final version.

## Conflict of Interest Statement

The authors declare that the research was conducted in the absence of any commercial or financial relationships that could be construed as a potential conflict of interest.
